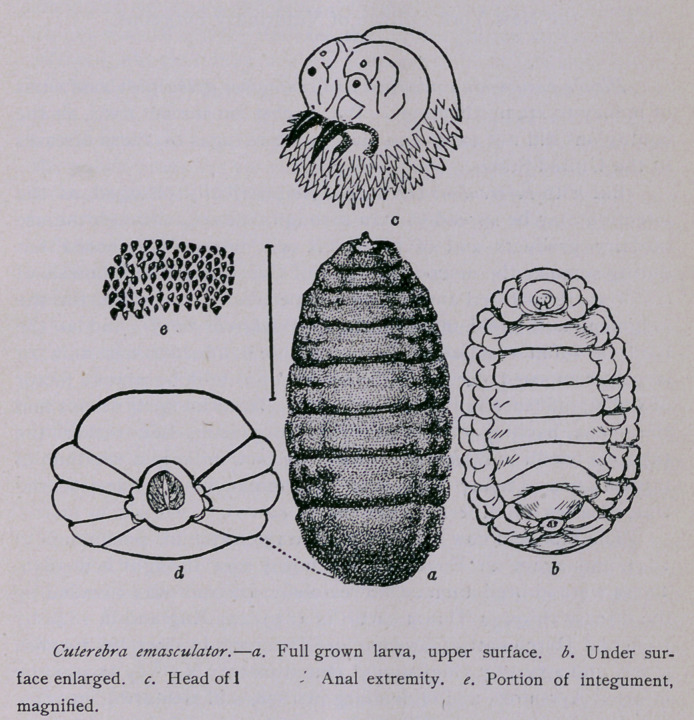# On the Emasculating Bot-fly

**Published:** 1894-03

**Authors:** 


### ON THE EMASCULATING BOT-FLY.

In the last issue of the *Veterinary Journal* (page 108),* allusion was made to the discovery of the larva of a bot-fly in the scrotum of a dog, and that it had been identified as belonging to the genus *Cuterebra*, individuals of which are known to infest squirrels, hares and mice. This is, we believe, the first mention of this cuticolous larva infesting one of the domesticated species, and now that attention is directed to the discovery, it is not unlikely that it may be found in some of the other species.

In Neumann's "Classical Treatise on the Parasites and Parasitic Diseases of the Domesticated Animals" (English translation, p. 46), it is stated that of the thirteen genera into which Brauer divided the Œstridæ, only four—*Hypoderma*, *Dermatobia*, *Cuterebra*, and *Œstromyia*—were cuticole larvæ, and that of these four genera

*From JOURNAL OF COMPARATIVE MEDICINE AND VETERINARY ARCHIVES, Vol. xiv, No. 6, page 379.

the first two alone develop as larvæ beneath the skin of domestic animals in Europe or other parts of the world, and were, therefore, only those to be described in the above-named book.

In consequence of the discovery of the Cuterebra larvæ in the scrotum of a dog in Canada, this statement will have to be modified, and it is possible that these larval insects may be found infesting the scrotum of other domesticated creatures.

In view of this possibility, it may be interesting and useful to ascertain what is known of this emasculating bot.

It appears that a Dr. Fitch, in his "Fourth New York Report," published nearly half a century ago, gave a long and particular account of it, and this at the time attracted great attention. That a bot-fly existed which, according to his statements, apparently bred only in the testicles of "chipmunks" or "gophers" and squirrels, was looked upon as a remarkable circumstance. He succeeded in rearing only one adult, which issued about July 29th, 1857, from earth in a jar in which the larva had been placed on September 1st, 1856. So far as is known, this is the only adult of the species which has ever been reared.

Dr. Fitch, in his interesting and careful description of the different stages in the development of the insect, gave the species the name of *Cuterebro emasculator* from the larval habit which he supposed to be characteristic. He mentions the fact that hunters in the vicinty of Lakeville, New York, where the first specimen sent him was found, had long been familiar with the fact at least one-half of the male gray squirrels shot in that vicinity were found to be castrated, and that it was the opinion of these men that the mutilation was caused by the squirrels seizing and biting out the testicles of their comrades. In support of this idea he gave the testimony of Mr. Hurst, taxidermist of the New York State Cabinet of Natural History, who claimed to have seen a half-dozen red squirrels unite in mastering a gray one and castrating it. Dr. Fitch queried whether the bot-fly might not be attracted by the wound so made, if this habit proved common, but concluded that the object of the joint attack of several squirrels upon one was rather to kill the grub which was engaged in emasculating the creature.

Since Fitch's publication the fly and its larva have not received much notice from entomologists, and it is not until 1889 that it is again specially referred to in one of the numbers of an excellent serial* issued by the Entomological Division of the

* "Insect Life," Vol. I, No. 7. We owe our best thanks to Dr. Salmon of the United States Bureau of Animal Industry, for a copy of this serial.

United States Department of Agriculture, to which we are indebted for the particulars now given on the subject.

It seems that there is some doubt as to whether Fitch's species will prove correct. Brauer, in his monograph on the Œstridæ (page 232), quotes Fitch's description at length, and asserts that he cannot separate the species from *Cuterebra scutellaris* (Löw), a North American species, the habits of which do not seem to be known. The finding of the larva in the scrotum of a dog in Canada, would almost lead one to believe in the identity of Fitch and Löw's insect.

The serial above alluded to remarks, that if this interesting insect has not attracted much attention of late years from entomologists, it has **not** failed to be noticed by zoologists and taxidermists; although the writer of the article is not aware that observations have been published. The following statement was written at his request by Dr. Merriam, the ornithologist of the Department of Agriculture, as he had made notes some time previously on the abundance of the insect in New York State :

" In reply to your inquiry concerning the occurrence of Cuterebræ in squirrels, I would state that during many years collecting in the Adirondack region of northern New York, particularly along its western border, in the Black River Valley, I frequently found Cuterebræ in or near the scrotum in the Gray Squirrel (*Sciurns Caroliuensis leucotis*), Red Squirrel (*Sciurus Hudsonius*), and Chipmunk (*Tamias striatus lysteri*). I have observed the same thing at East Hampton, Massachusetts, and in other localities. The most extraordinary instance of the prevalence of this disgusting parasite that has fallen under my observation was at the south end of Lake Champlain, New York, in October, 1885. On the 7th and 9th of that month I killed more than fifty chipmunks within a few miles of old Fort Ticonderoga, and on the rocky side hill behind the town of Whitehall. Of these a very large percentage—I think fully one-half—were infested with "wabbles" (Cuterebræ). More females than males were thus afflicted. The wabbles* were usually situated near the median line, and anywhere from the umbilical region to the genitals. In a few cases they were in the axilla, and in one or two instances in the upper part of the foreleg. In a number of individuals two Cuterebræ were found, and in a few cases as many as three. Dr. A. K. Fisher tells me that he collected a number of chipmunks about the south end of Lake George, Warren County, N. Y., during the latter part of August and 1st of September, 1882, a considerable proportion of which were infested with Cuterebræ. As many as three were found, in different stages of development, in one animal. A gray squirrel killed at Sing Sing, Westchester County, N. Y., contained a Cuterebra in the left pectoral region."

The object in publishing the date in the periodical referred to, was to introduce for the first time careful figures of the full-

---

* Our English "Warbles."

grown larva, obtained from a recently dead chipmunk which had been put into a box. The larva or maggot was found in a corner of the box, it having escaped from a "swelling" under the animal's body; the tumor was about an inch long and of the shape of a mulberry, and there appeared to be a natural opening at its apex more than a sixteenth of an inch in diameter, with a tinge of dark liquid about it.

*Cuterebra emasculator.*—*a.* Full grown larva, upper surface. *b.* Under surface enlarged. *c.* Head of l[illegible] Anal extremity. *e.* Portion of integument, magnified.

—*The Veterinary Journal.*

## Figures and Tables

**Figure f1:**